# Progress of Surgery

**Published:** 1901-07-27

**Authors:** 


### Progress of Surgery.

### SURGERY OF THE BLADDER AND URETERS.

**Ureteral Calculus Simulating Vesical Calculus.**—Francis J. Steward[1] recently related a case of this kind before the Clinical Society. The patient, a man 24 years of age, had suffered from painful micturition and hæmaturia for three years, the attacks gradually getting more severe. A little pus was found in the urine before operation. The pain with micturition was severe and situated in the perineum and at the end of the penis. Radiographs were taken and the bladder sounded with negative results. The bladder was opened supra-pubically after inflating with air, and a stone felt impacted in the right ureter two inches from its vesical orifice. The stone could not be moved down, so the wound was closed. Three weeks later the abdomen was opened through the right linea semilunaris. The stone was then pushed up the ureter to just above the common iliac artery and removed by incising the ureter. The ureter was closed by a continuous silk suture of No. 0 Chinese twist passing only through the outer coats, and the peritoneum was sewn over it. A gauze drain was left passing to the sutured part of the ureter. Uncomplicated recovery followed. Steward remarked that the chief point of interest was that the symptoms pointed to vesical calculus and were at no time unilateral. The air distension rendered the supra-pubic cystotomy very easy, the peritoneum being well lifted up, and the absence of the usual flooding of the wound on opening the bladder was a great advantage. The gauze drain had been put in from the front to avoid turning the patient over and cleansing the skin, as he was already exhausted.

**Total Ureterectomy.**—Willy Meyer,[2] New York, has recorded a case of this procedure. The patient had had the left kidney removed for pyelonephrosis several years before, and on another occasion later the bladder had been drained both above the pubes and through the perineum. The patient suffered from left-sided pain passing into the extremity, and frequent micturition. It was impossible to examine the bladder with the cystoscope owing to the existence of a valve in the posterior urethra, such as repeatedly occurred after prolonged perineal drainage. Two specimens of urine were therefore collected, one before and the other after massage of the ureter on the affected side. There had been such a marked difference in the two specimens that the diagnosis was established. The operation was very difficult because the ureter had been cut off low down at the previous operation.

**Renal Colic Associated with the Passage of Casts of the Ureter.**—J. A. Henton White[3] has recorded a case of this affection. The patient, a woman of over 60, had suffered for twenty years from attacks of agonising pain in the left loin. When first seen by White, fifteen months before, the attacks were occurring six or seven times a week. Owing to rigidity the kidney could not be felt. There was great tenderness below the left lower ribs posteriorly, and along the course of the ureter. The urine, when examined during an attack, was slightly acid with a smoky tinge, and contained a distinct cloud of albumen, and a few blood cells. The most remarkable objects, however, were a number of semi-transparent mucoid strings about an inch long. For fifteen months the patient had always noticed these strings after a bad attack of colic. Microscopically they were hollow, elongated, cylindrical bodies of clear mucus held together by a few threads of fibrin and containing a few small granules and the remains of a few epithelial cells which occasionally showed a transitional character. An alkaline mixture containing 2 grs. of potassium iodide taken three times a day gave great relief. On leaving off taking this the patient had again bad attacks of colic. An acid mixture containing belladonna was then given, but this did her no good. The alkaline mixture again gave relief and reduced the number of attacks to about one a month. A case in which ureteral casts were passed observed by Prof. von Jaksch is quoted. In that case, however, the patient had previously passed a renal calculus. Von Jaksch compares the condition to Curschmann's spirals, found in certain forms of bronchitis, and to cases of membranous colitis. Fenwick, of New Zealand, has related a case in which a membranous cast of the gall-bladder and duct produced attacks of biliary colic. White thinks it very probable that a renal calculus is present in his case, and that this, by irritating the renal plexus, produces nutritive disorders of the ureteral mucous membrane.

**Reflex Passage of Vesical Contents into the Ureters.**—Filiberto Jacobelli,[4] from the results of experiments upon animals, concludes that the descending flow of urine constitutes the true protection of the ureters. When this is abolished there is a rapid immigration of bacteria. Vesical stasis is not essential to this invasion. Stasis has an indirect action, but the ascent of the micro-organisms is not due to diffusion along a column of fluid, as Guyon holds, but to motion inherent in the bacteria themselves. This motion is opposed by the descending current, but if the latter be interfered with—for instance, by a calculus—the ascent of bacteria is greatly facilitated. Possibly the origin of pyonephrosis from calculus may be accounted for in this way.

[1] *Vide* the Lancet, April, 1901. [2] Med. Rec., Dec., 1900. [3] Brit. Med. Jour., Jan., 1901. [4] *Vide* Abst. Med. Rec., Feb., 1901.

